# The Association of Aging with White Matter Integrity and Functional Connectivity Hubs

**DOI:** 10.3389/fnagi.2016.00143

**Published:** 2016-06-15

**Authors:** Albert C. Yang, Shih-Jen Tsai, Mu-En Liu, Chu-Chung Huang, Ching-Po Lin

**Affiliations:** ^1^Division of Interdisciplinary Medicine and Biotechnology, Beth Israel Deaconess Medical Center/Harvard Medical SchoolBoston, MA, USA; ^2^Department of Psychiatry, Taipei Veterans General HospitalTaipei, Taiwan; ^3^Division of Psychiatry, School of Medicine, National Yang-Ming UniversityTaipei, Taiwan; ^4^Center for Dynamical Biomarkers and Translational Medicine, National Central UniversityChungli, Taiwan; ^5^Institute of Brain Science, National Yang-Ming UniversityTaipei, Taiwan

**Keywords:** aging, diffusion tensor imaging, functional connectivity density mapping, compensatory hypothesis

## Abstract

Normal aging is associated with reduced cerebral structural integrity and altered functional brain activity, yet the association of aging with the relationship between structural and functional brain changes remains unclear. Using combined diffusion tensor imaging (DTI) and functional magnetic resonance imaging (fMRI) modalities, we hypothesized that aging-related changes in white matter integrity (i.e., fractional anisotropy) was associated with the short- or long-range functional connectivity density (FCD) in hub regions. We tested this hypothesis by using a healthy aging cohort comprised of 140 younger adults aged 20–39 years and 109 older adults aged 60–79 years. Compared with the younger group, older adults exhibited widespread reductions in white matter integrity with selective preservation in brain stem tracts and the cingulum connected to the hippocampus and cingulate cortex, whereas FCD mapping in older adults showed a reduced FCD in the visual, somatosensory, and motor functional networks and an increased FCD in the default mode network. The older adults exhibited significantly increased short- or long-range FCD in functional hubs of the precuneus, posterior, and middle cingulate, and thalamus, hippocampus, fusiform, and inferior temporal cortex. Furthermore, DTI-fMRI relationship were predominantly identified in older adults in whom short- and long-range FCD in the left precuneus was negatively correlated to structural integrity of adjacent and nonadjacent white matter tracts, respectively. We also found that long-range FCD in the left precuneus was positively correlated to cognitive function. These results support the compensatory hypothesis of neurocognitive aging theory and reveal the DTI-fMRI relationship associated with normal aging.

## Introduction

Aging is associated with remarkable changes in brain structure and function. Studies have revealed that, compared with younger adults, older people exhibit reduced brain size, and increased ventricular volume (Scahill et al., 2003), loss of neuronal body or synaptic density (Morrison and Hof, 1997), loss of gray matter intensity (Good et al., 2001), or reduced white matter integrity (Pagani et al., 2008). However, research with blood oxygen level dependent (BOLD) signals from functional magnetic resonance imaging (fMRI; Fox and Raichle, 2007) has identified a disturbed functional connectivity associated with aging (see Ferreira and Busatto, 2013 for reviews). Nevertheless, the relationship between structural and functional brain changes associated with aging remains under explored (see Minati et al., 2007; Bennett and Rypma, 2013 for reviews).

Because transmission of neuronal signals relies on physical connections between a myriad of neurons via axons and synapses, neural activity is thought to be modulated through structural connections (Koch et al., 2002; Boorman et al., 2007; Greicius et al., 2009). Furthermore, the quality of structural connections, or white matter integrity, has been suggested to influence functional connectivity of gray matter brain regions (Bennett and Rypma, 2013). In this context, combining structural and fMRI techniques is appropriate for examining the relationship between brain structure and functional changes associated with aging. Whereas, diffusion tensor imaging (DTI) can be used to quantify white matter structural integrity, such as fractional anisotropy (FA) value, resting-state BOLD fMRI can reveal functional dependency among different brain regions.

Previous studies examining the effect of aging on the relationship between structural integrity and functional connectivity have suggested that patterns vary between younger and older adults (Bennett and Rypma, 2013). White matter integrity has been positively correlated to functional activity in younger adults (Forstmann et al., 2008; Kim and Whalen, 2009; Van Eimeren et al., 2010), but negatively correlated to functional activity in older adults (Persson et al., 2006; Madden et al., 2007). However, other studies have shown both patterns (Baird et al., 2005; Koch et al., 2010) or opposite directions (Putnam et al., 2008; De Chastelaine et al., 2011) of DTI-fMRI correlation, relative to the findings of previously mentioned studies. Studies on aging regarding the DTI-fMRI relationship have predominantly been based on various cognitive tasks (Bennett and Rypma, 2013), and the effect of aging on the relationship between structural integrity and resting-state functional connectivity is unclear. Such an investigation could facilitate clarifying the neurocognitive mechanism in aging, particularly in the default mode network (DMN) brain regions.

Therefore, this study investigated the association of aging with structural integrity and resting-state functional connectivity in a healthy aging cohort. We focused on highly connected hub regions of functional networks based on functional connectivity density (FCD) mapping (Tomasi and Volkow, 2010, 2012a). This study tested the hypotheses that aging-related changes in structural integrity was associated with alterations in short- or long-range functional connectivity in hub regions, and that this association predominantly occurs in older adults to compensate for the structural decline associated with normal aging.

## Materials and methods

### Participants

The study cohort comprised 249 Han Chinese participants recruited from communities in Northern Taiwan. Among them, 140 participants aged 20–39 years comprised the younger group (mean: 28.5 ± 4.9 years; male/female: 67/73), and 109 participants aged 60–79 years comprised the older group (mean: 67.4 ± 6.2 years; male/female: 52/57). The study constituted a continuing effort of the Healthy Aging Project (Yang et al., 2013, 2014), and received approval from the Institutional Review Board of Taipei Veterans General Hospital. Each participant gave written informed consent and was evaluated by a trained research assistant using the Mini-International Neuropsychiatric Interview to exclude the presence of Axis I psychiatric disorders (Sheehan et al., 1998). All participants were assessed for cognitive function, using the Mini-Mental State Examination (MMSE; Folstein et al., 1975) and the Wechsler Digit Span Task (Wechsler, 1997). Older participants were further assessed using the Clinical Dementia Rating Scale (CDR) (Hughes et al., 1982) to exclude dementia (CDR > 0). Overall exclusion criteria for all participants were as follows: (a) presence of dementia; (b) presence of Axis I psychiatric disorders, such as schizophrenia, bipolar disorders, or unipolar depression; and (c) a history of neurological conditions, such as head injury, stroke, or Parkinson's disease. Demographic characteristics of age groups are shown in Table 1.

**Table 1:** **Demographic characteristics in different age groups**.

**Variables**	**Younger (*N* = 140)**	**Older (*N* = 109)**	***t or x***	***P***
Age, year	28.5 ± 4.9	67.5 ± 6.2	−54.9	< 0.001
Sex, male	67 (47.9)	52 (47.7)	0.01	n.s.
Handedness, right	133 (95.0)	105 (96.3)	0.04	n.s.
Mini mental state examination	29.2 ± 1.1	27.6 ± 2.5	6.8	< 0.001
Digit forward task	15.3 ± 1.4	13.2 ± 2.2	8.7	< 0.001
Digit backward task	10.8 ± 2.9	6.9 ± 3.3	9.8	< 0.001

Categorical data are given as number (%). n.s., non-significant.

### Image acquisition

We performed fMRI scanning at National Yang-Ming University, using a 3.0T Siemens MRI scanner (Siemens Magnetom Tim Trio, Erlangen, Germany) equipped with a 12-channel head coil. The scanning protocol was consistent with our prior reports (Yang et al., 2013, 2014). For resting-state image scanning, T2^*^-weighted images with BOLD contrast were measured using a gradient echo-planar imaging (EPI) sequence (repetition time TR = 2500 ms, echo time TE = 27 ms, FoV = 200 mm, flip angle = 77°, matrix size = 64 × 64, voxel size = 3.44 × 3.44 × 3.40 mm). For each run, 200 EPI volume images were acquired along the AC–PC plane. For structural image scanning, whole-brain DTI images were acquired using a single-shot spin-echo EPI sequence in the axial-plane with the following parameters: repetition time/echo time = 11,000/104 ms; number of excitations = 3; matrix size = 128 × 128; field of view = 26 cm; slice thickness = 2.0 mm; 70 slices; *b*-value = 1000 s/mm^2^; 30 isotropic diffusion directions and three non-diffusion weighted T2 images.

### Structural and resting imaging processing

Resting BOLD image data were preprocessed and analyzed using SPM8 (Wellcome Department of Imaging Neuroscience, London, UK) implemented in MATLAB (Mathworks Inc., Sherborn, MA, USA). BOLD data were slice-timing corrected, realigned, and normalized into the standard stereotaxic space of the Montreal Neurological Institute (MNI) EPI template, and resampled to a 3-mm cubic voxel. Covariates of BOLD time series were regressed out before FCD analysis was performed, including the time courses of six head motions, white matter, and cerebrospinal fluid. No global signal regression was performed to avoid introducing distortions in the time series data (Murphy et al., 2009; Anderson et al., 2011). All participants included in this study exhibited a maximal displacement of < 1.5 mm at each axis and an angular motion of < 1.5° for each axis. The first five data points (12.5 s) in any BOLD time series were discarded because of the instability of initial MRI scanning, leaving 195 data points in the final data. Temporal low-pass filtering (0.01–0.08 Hz) was performed to reduce the influence of high-frequency noise from physiologic confounders.

Raw DTI images were preprocessed using the Functional Magnetic Resonance Imaging of the Brain (FMRIB) diffusion toolbox (Raz et al., 2007), part of the Functional Magnetic Resonance Imaging of the Brain software library (FSL; Morrison and Hof, 1997). The raw DTI image was corrected for the effects of head movement and eddy currents. A brain extraction tool was used to remove all non-brain parts of the image (Good et al., 2001). From these images, fractional anisotropy (FA) values were calculated by fitting a tensor model to the data at each voxel. The Tract-Based Spatial Statistics (TBSS, part of FSL) pipeline (Scahill et al., 2003) was then used to register and normalize FA images to the MNI standard space used to target the FMRIB58 FA standard-space image. A mean FA image was created and thinned to create a mean FA skeleton that represented the centers of all tracts common to the group. An FA threshold of 0.2 was used to exclude non-skeletal voxels (Scahill et al., 2003). The aligned FA data of each subject was then projected onto this skeleton, and the resulting data were subjected to subsequent statistical analyses.

### Functional connectivity density mapping

FCD mapping (Tomasi and Volkow, 2010, 2012a) was developed based on resting-state BOLD signals for mapping whole-brain short-range and long-range functional connectivity with high spatial resolution (3-mm isotropic), which allows identifying functional hubs (Tomasi and Volkow, 2010, 2011). Tomasi et al. defined three types of FCD measure: global FCD, short-range FCD, and long-range FCD.

For each gray matter voxel, global FCD was defined as the number of global functional connections, k(x_*i*_), determined through Pearson correlations between BOLD signals at voxel x_*i*_ and those in the remaining gray matter voxels using an arbitrary threshold *r* > 0.6 (Tomasi and Volkow, 2010). Short-range FCD was defined as local neighbors of voxel x_*i*_, which was computed using a growth algorithm to identify voxels that were adjacent to a voxel linked to x_*i*_ on a continuous path, and had connectivity strength *r* > 0.6 with voxel x_*i*_. The growth algorithm was repeated in an iterative manner until no new neighbors could be found.

In the final step, the long-range FCD of voxel x_i_ was computed by subtracting global FCD and short-range FCD. The calculation of FCD was computed for all gray matter voxels. The whole-brain short- and long-range FCD maps were further normalized by the whole-brain mean FCD, and were spatially smoothed with an 8-mm Gaussian kernel in SPM8 to minimize differences in the functional anatomy of the brain across subjects (Tomasi and Volkow, 2012a).

### Statistical analysis

**Figure 1:**
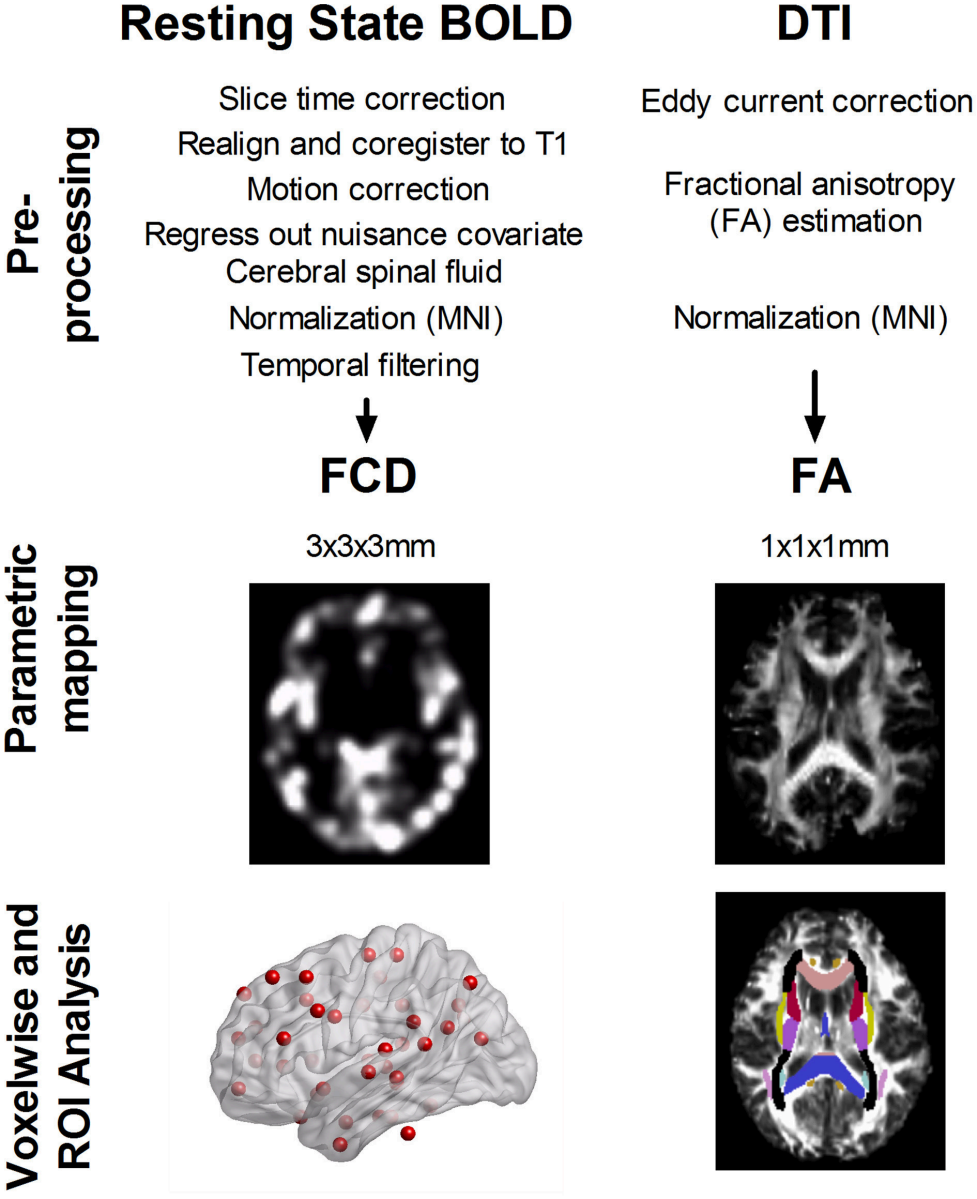
**Analysis flow of resting-state blood oxygen level dependent (BOLD) signals and diffusion tensor images (DTI)**. Both data were preprocessed according to the standard procedure. Short- and long-range functional connectivity density (FCD) was calculated from resting-state BOLD signals, and fractional anisotropy (FA) was determined from DTI data. We analyzed the between age-group difference in FCD and FA, using ROI analysis over 42 functional hubs and 48 white matter tracts. Furthermore, we determined the correlation between FA of 48 white matter tracts and FCD value at 42 functional hubs in younger and older adults.

Figure 1 illustrates the analysis flow of image analyses. Statistical analyses of parametric imaging data were conducted using MATLAB. First, regional differences in whole-brain parametric mapping (i.e., short-range and long-range FCD and DTI-FA) between age groups were examined using the general linear model. Second, we determined the anatomical regions of interest (ROIs) based on 48 white matter tract labels defined in the ICBM-DTI-81 atlas developed at Johns Hopkins University (Minati et al., 2007; Bennett and Rypma, 2013). The averaged FA of each white matter tract was calculated by overlaying the group-specific white matter skeleton and the ICBM-DTI-81 atlas with a specific white matter labeling (Van Eimeren et al., 2010). The strength of the short- and long-range FCD were similarly calculated from 42 functional hubs identified previously (Tomasi and Volkow, 2012a). We used a cubic mask containing 27 voxels centered on hub coordinates to extract the average FCD values. A Student's *t*-test was used to assess between-group difference in ROI measures. These FCD and FA measures were additionally used to assess the correlation between FA and FCD values derived from gray and white matter ROIs at the group level, respectively. This approach determined the extent to which the FCD of the gray matter hub can be explained in a group of subjects by using the mean FA value of a given white matter tract, controlling for the effect of age and sex. Hence, we performed a partial correlation to control for age and sex and conducted the analyses separately in the younger and older group. Bonferroni correction for multiple comparisons was used for between-group difference in FA, short-range, or long-range FCD measures. Specifically, a *P* < 0.001 was used to assess between-group difference in FA measures (i.e., 0.05/48 tests), and a *P* < 0.0012 was used to assess between-group difference in short- or long-range FCD measures (i.e., 0.05/42 tests). For partial correlations between FA values and short- or long-range FCD measures, any correlation with an uncorrected *P* < 0.001 was reported and was further corrected by false discovery rate (FDR) method for multiple correlation tests (i.e., 48 white matter tracts multiplied by 42 functional hubs).

## Results

### The association of age with short- and long-range functional connectivity density

**Figure 2:**
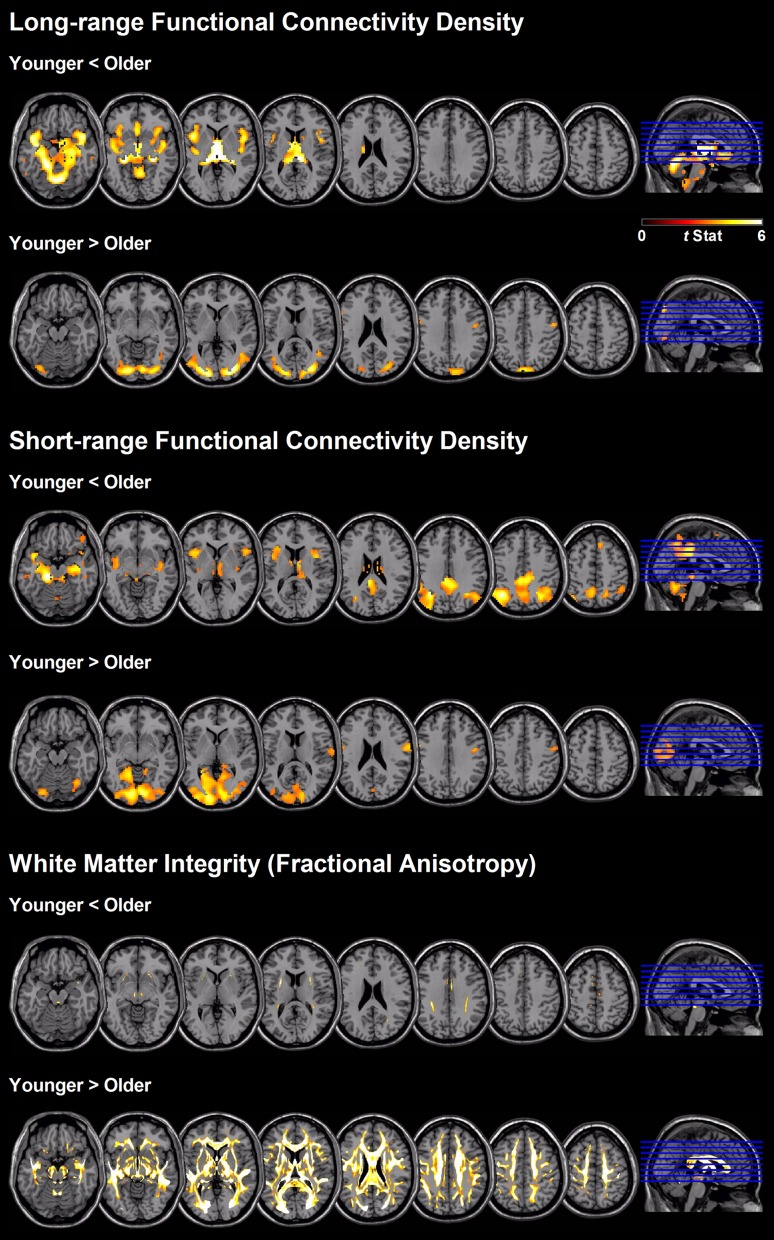
**Regional differences between age-groups in short- and long-range FCD, as well as white matter integrity based on fractional anisotropy value**.

Figure 2 shows the regions with significant between-age-group differences in short- and long-range FCD mapping. Compared with younger adults, older subjects exhibited a significant reduction in long-range FCD in the lingual, calcarine, postcentral, and precentral cortex, and a significant increase in long-range FCD in the cerebellum, hippocampus, thalamus, insula, caudate, amygdala, fusiform, and anterior cingulate cortex. However, older subjects exhibited a significant reduction in short-range FCD in the lingual, calcarine, postcentral and precentral gyrus, and a significant increase in short-range FCD in the cerebellum, precuneus, posterior cingulate, hippocampus, thalamus, insula, angular gyrus, and supplementary motor area, compared with younger adults (only qualitative results were reported because of large clusters).

Table 2 presents the between-age-group difference in FCD based on 42 functional hub regions. The results are similar to the voxel-wise analysis presented in Figure 2, except that no hub region was found to have significantly reduced FCD in older adults compared with younger people. Older people had increased long-range FCD in hubs of the thalamus (*t* = −6.0), left and right hippocampus (*t* = −4.7 and −3.9, respectively), left fusiform gyrus (*t* = −5.2 and −3.6, respectively), and right cerebellum (*t* = −3.5), as well as increased short-range FCD in hubs of the left precuneus (*t* = −3.3), left posterior cingulate (*t* = −3.8), left inferior parietal (*t* = −4.1), right angular (*t* = −3.7), left hippocampus (*t* = −3.4), left fusiform (*t* = −4.0), left middle cingulate (*t* = −3.2), and inferior temporal cortex (*t* = −3.5; all *p* < 0.001).

**Table 2:** **Between-group difference in long-range and short-range functional connectivity density in 42 functional hubs**.

**No**.	**Brain region**	**BA**	**MNI coord. (mm)**			**LFCD vs. age**		**Direction**	**SFCD vs. age**		**Direction**
			***x***	***y***	***z***	***t***	***P***		***t***	***P***	
1	Precuneus R	7	3	−63	36	−0.3	n.s.		−2.9	n.s.	
2	Precuneus L	23	−3	−57	24	−0.1	n.s.		−3.3	< 0.001	Y < O
3	Pos. cingulate L	23	−3	−39	27	−0.2	n.s.		−3.8	< 0.001	Y < O
4	Inf. parietal L	7	−36	−69	48	0	n.s.		−4.1	< 0.001	Y < O
5	Mid. orbital frontal	11	0	54	−9	0.3	n.s.		−0.7	n.s.	
6	Sup. medial frontal	32	0	54	18	2.1	n.s.		0.2	n.s.	
7	Sup. medial frontal	9	0	51	42	−0.5	n.s.		−0.1	n.s.	
8	Ant. cingulate R	32	6	18	39	−0.7	n.s.		−0.4	n.s.	
9	Mid. frontal R	45	45	45	6	1.2	n.s.		0.2	n.s.	
10	Supramarginal R	40	63	−33	36	−0.8	n.s.		−2.9	n.s.	
11	Angular R	39	48	−66	30	−1.9	n.s.		−3.7	< 0.001	Y < O
12	Sup. frontal L	9	−21	36	51	1.2	n.s.		−0.6	n.s.	
13	Mid. frontal L	9	−42	18	51	1.1	n.s.		−0.4	n.s.	
14	Precentral L	44	−48	12	33	2.0	n.s.		0.7	n.s.	
15	Inf. frontal R	47	36	30	3	−1.6	n.s.		−1.5	n.s.	
16	Mid. frontal R	46	33	45	24	0.5	n.s.		−0.8	n.s.	
17	Nucleus accumbens R	25	15	12	−12	1.3	n.s.		−2.0	n.s.	
18	Nucleus accumbens L	25	−12	9	−9	2.1	n.s.		−0.5	n.s.	
19	Rectus	11	0	21	−24	−0.7	n.s.		−1.7	n.s.	
20	Inf. frontal L	45	−42	30	18	0.3	n.s.		0.2	n.s.	
21	Caudate R		9	15	6	−1.0	n.s.		−1.9	n.s.	
22	Post−central L	4	−24	−30	63	−0.7	n.s.		0.0	n.s.	
23	Pre−central	6	−24	−15	63	−0.7	n.s.		−1.6	n.s.	
24	Post−central R	3	42	−21	51	−0.4	n.s.		0.9	n.s.	
25	Thalamus R		12	−24	3	−6.0	< 0.001	Y < O	−2.5	n.s.	
26	Hippocampus R	27	21	−30	−6	−3.9	< 0.001	Y < O	−1.3	n.s.	
27	Fusiform L	37	−24	−36	−33	−5.2	< 0.001	Y < O	−2.6	n.s.	
28	Hippocampus L	27	−18	−30	−3	−4.7	< 0.001	Y < O	−3.4	< 0.001	Y < O
29	Fusiform L	36	−36	0	−39	−3.6	< 0.001	Y < O	−4.0	< 0.001	Y < O
30	Insula R	13	36	−15	6	−2.1	n.s.		−2.1	n.s.	
31	Cerebellum R		36	−3	−27	−3.5	< 0.001	Y < O	−3.0	n.s.	
32	Pons R		12	−27	−24	−2.9	n.s.		−3.1	n.s.	
33	Heschl L	13	−42	−24	12	−2.3	n.s.		−0.5	n.s.	
34	Sup. temporal L	22	−63	−15	0	1.7	n.s.		2.5	n.s.	
35	Mid. cingulate L	24	−9	3	30	−1.8	n.s.		−3.2	< 0.001	Y < O
36	Mid. temporal L	21	−45	−45	15	−0.4	n.s.		−0.9	n.s.	
37	Sup. temporal R	41	39	−33	15	−0.8	n.s.		0.7	n.s.	
38	Fusiform R	37	39	−48	−18	1.3	n.s.		1.1	n.s.	
39	Mid. cingulate R	23	12	−12	36	1.5	n.s.		−3.0	n.s.	
40	Inf. temporal R	20	42	−21	−24	−2.8	n.s.		−3.5	< 0.001	Y < O
41	Sup. temporal R	22	69	−18	6	0.9	n.s.		1.9	n.s.	
42	Calcarine L	18	−18	−75	18	1.4	n.s.		0.1	n.s.	

n.s., non-significant. All results had P < 0.0012 using Bonferroni corrections for multiple comparisons (i.e., 0.05 divided by 42 tests for LFCD or SFCD).

### The association of age with white matter integrity

Older people showed a widespread reduction in white matter integrity measured according to FA value, compared with younger adults (Figure 2 and Table 3). The most affected region was the column and body of the fornix (*t* = 13.5), followed by the genu of the corpus callosum (*t* = 9.9), anterior and superior corona radiate (both *t* = 9.6), left and right fornix cres stria terminalis (*t* = 9.4 and 9.2, respectively), tapetum (*t* = 9.2), and body of the corpus callosum (*t* = 9.2). The unaffected white matter tracts were the corticospinal tract, medial lemniscus, part of the cerebellum peduncle, and the cingulum involved in the hippocampus and cingulate gyrus.

**Table 3:** **Between-group difference in fractional anisotropy in 48 white matter tracts**.

**No**.	**White Matter Tract**	**Younger**	**Older**	***t***	***P***	**Direction**
1	Mid. cerebellar peduncle	0.33 ± 0.13	0.29 ± 0.11	2.4	n.s.	
2	Pontine crossing tract	0.36 ± 0.12	0.33 ± 0.11	1.7	n.s.	
3	Genu of corpus callosum	0.60 ± 0.02	0.53 ± 0.07	9.9	< 0.001	Y > O
4	Body of corpus callosum	0.60 ± 0.02	0.53 ± 0.09	9.2	< 0.001	Y > O
5	Splenium of corpus callosum	0.67 ± 0.02	0.60 ± 0.11	7.2	< 0.001	Y > O
6	Fornix column and body of fornix	0.36 ± 0.06	0.24 ± 0.08	13.5	< 0.001	Y > O
7	Corticospinal tract R	0.45 ± 0.10	0.45 ± 0.13	0	n.s.	
8	Corticospinal tract L	0.40 ± 0.10	0.40 ± 0.11	−0.2	n.s.	
9	Medial lemniscus R	0.43 ± 0.15	0.40 ± 0.16	1.7	n.s.	
10	Medial lemniscus L	0.40 ± 0.18	0.38 ± 0.16	1.0	n.s.	
11	Inf. cerebellar peduncle R	0.36 ± 0.13	0.26 ± 0.14	5.7	< 0.001	Y > O
12	Inf. cerebellar peduncle L	0.29 ± 0.16	0.23 ± 0.16	3.2	n.s.	
13	Sup. cerebellar peduncle R	0.48 ± 0.06	0.43 ± 0.11	3.9	< 0.001	Y > O
14	Sup. cerebellar peduncle L	0.45 ± 0.06	0.43 ± 0.09	1.4	n.s.	
15	Cerebral peduncle R	0.62 ± 0.02	0.55 ± 0.12	6.6	< 0.001	Y > O
16	Cerebral peduncle L	0.55 ± 0.02	0.50 ± 0.10	5.7	< 0.001	Y > O
17	Ant. limb of int. capsule R	0.51 ± 0.02	0.46 ± 0.08	6.0	< 0.001	Y > O
18	Ant. Limb of Int. Capsule L	0.52 ± 0.02	0.46 ± 0.08	7.5	< 0.001	Y > O
19	Post. limb of int. capsule R	0.64 ± 0.02	0.58 ± 0.12	6.2	< 0.001	Y > O
20	Post. limb of int. capsule L	0.52 ± 0.01	0.47 ± 0.08	6.6	< 0.001	Y > O
21	Retrolenticular R	0.53 ± 0.02	0.48 ± 0.08	6.8	< 0.001	Y > O
22	Retrolenticular L	0.46 ± 0.02	0.43 ± 0.07	5.7	< 0.001	Y > O
23	Ant. corona radiata R	0.41 ± 0.02	0.37 ± 0.04	2.0	< 0.001	Y > O
24	Ant. corona radiata L	0.43 ± 0.03	0.38 ± 0.05	9.6	< 0.001	Y > O
25	Sup. corona radiata R	0.47 ± 0.02	0.43 ± 0.06	7.4	< 0.001	Y > O
26	Sup. corona radiata L	0.45 ± 0.02	0.40 ± 0.06	9.6	< 0.001	Y > O
27	Pos. corona radiata R	0.42 ± 0.02	0.40 ± 0.06	5.0	< 0.001	Y > O
28	Pos. corona radiata L	0.46 ± 0.02	0.41 ± 0.06	8.6	< 0.001	Y > O
29	Post. thalamic radiation R	0.51 ± 0.04	0.45 ± 0.07	7.9	< 0.001	Y > O
30	Post. thalamic radiation L	0.48 ± 0.05	0.40 ± 0.09	9.1	< 0.001	Y > O
31	Sagittal stratum R	0.45 ± 0.07	0.34 ± 0.13	9.1	< 0.001	Y > O
32	Sagittal stratum L	0.39 ± 0.06	0.32 ± 0.10	7.6	< 0.001	Y > O
33	External capsule R	0.39 ± 0.02	0.34 ± 0.06	8.8	< 0.001	Y > O
34	External capsule L	0.37 ± 0.01	0.34 ± 0.05	5.6	< 0.001	Y > O
35	Cingulum cingulate gyrus R	0.54 ± 0.03	0.50 ± 0.08	5.7	< 0.001	Y > O
36	Cingulum cingulate gyrus L	0.36 ± 0.02	0.34 ± 0.05	3.0	n.s.	
37	Cingulum hippocampus R	0.34 ± 0.03	0.32 ± 0.07	3.1	n.s.	
38	Cingulum hippocampus L	0.31 ± 0.04	0.29 ± 0.08	2.8	n.s.	
39	Fornix cres stria terminalis R	0.50 ± 0.02	0.42 ± 0.09	9.2	< 0.001	Y > O
40	Fornix cres stria terminalis L	0.43 ± 0.02	0.37 ± 0.08	9.4	< 0.001	Y > O
41	Sup. longitudinal fasciculus R	0.46 ± 0.02	0.43 ± 0.07	6.0	< 0.001	Y > O
42	Sup. longitudinal fasciculus L	0.43 ± 0.02	0.41 ± 0.06	3.9	< 0.001	Y > O
43	Sup. fronto occipital fasciculus R	0.47 ± 0.03	0.42 ± 0.09	6.3	< 0.001	Y > O
44	Sup. fronto occipital fasciculus L	0.38 ± 0.03	0.32 ± 0.07	9.0	< 0.001	Y > O
45	Uncinate fasciculus R	0.39 ± 0.03	0.37 ± 0.10	2.7	n.s.	
46	Uncinate fasciculus L	0.38 ± 0.03	0.34 ± 0.07	5.3	< 0.001	Y > O
47	Tapetum R	0.52 ± 0.04	0.48 ± 0.07	5.5	< 0.001	Y > O
48	Tapetum L	0.55 ± 0.03	0.47 ± 0.09	9.2	< 0.001	Y > O

n.s., non-significant. All results had P < 0.001 using Bonferroni corrections for multiple comparisons (i.e., 0.05 divided by 48 tests).

### Relationship between functional connectivity and white matter integrity

**Figure 3:**
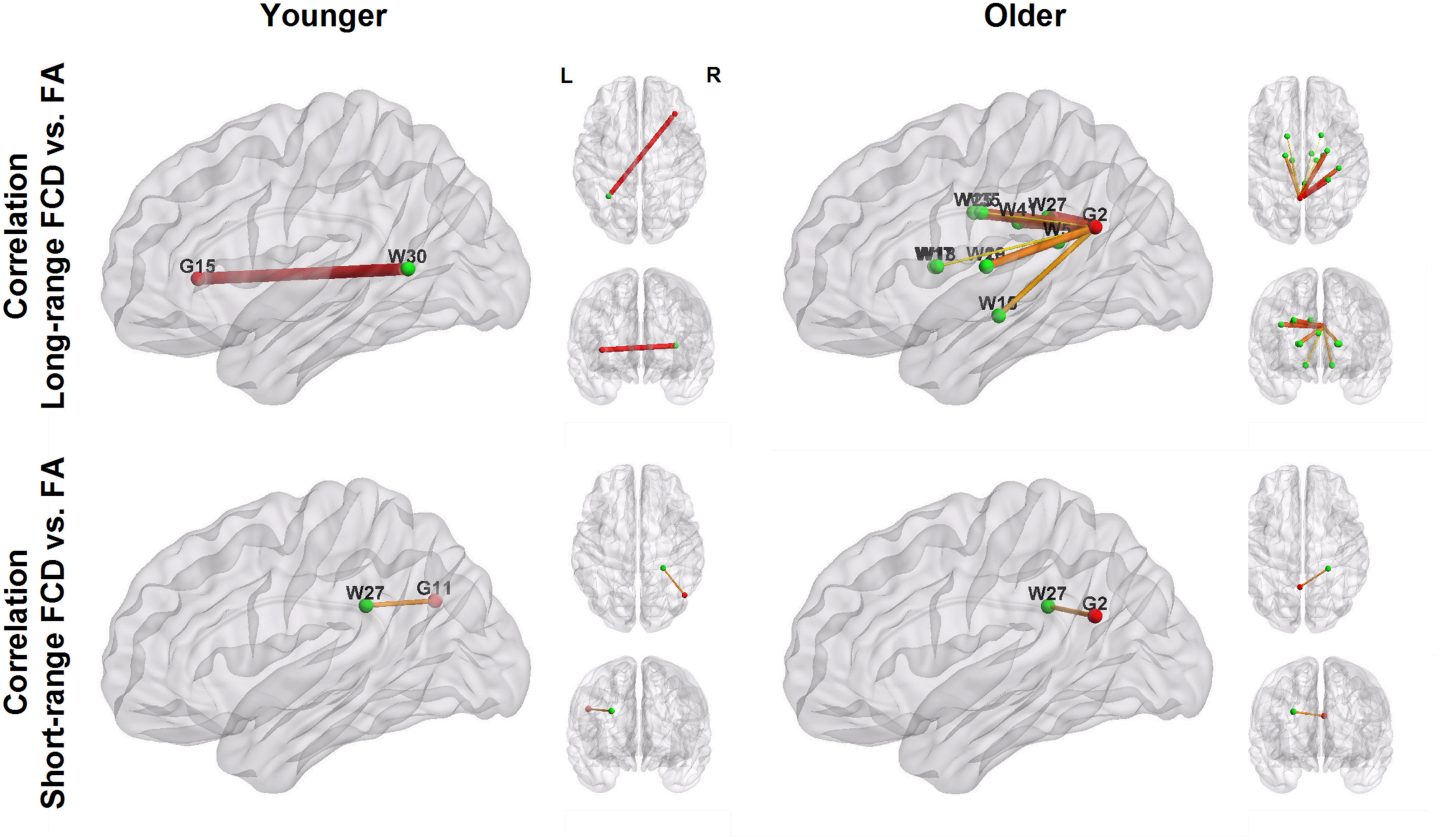
**Patterns of correlation between white matter integrity (green nodes; labeled with W) and FCD in hub regions (red nodes; labeled with G)**. The number shown in the node labels corresponds to the brain region order listed in Table 2 (G) and Table 3 (W). For example, G2, left precuneus; G11, right angular, G15: right inferior frontal. W27, right posterior corona radiata; W30, left posterior thalamic radiation.

Figure 3 and Table 4 show the significant correlation pairs between the FA value of white matter tracts and the FCD of functional hubs. Long-range FCD was generally correlated to the structural integrity of nonadjacent white matter tracts, whereas short-range FCD was correlated to the structural integrity of adjacent white matter tracts. In younger adults, long-range FCD in the right inferior frontal cortex was correlated negatively to the left posterior thalamic radiation (*r* = −0.29), and short-range FCD in the right angular gyrus was correlated positively to the right posterior corona radiata (*r* = 0.29).

**Table 4:** **Significant correlations of white matter integrity with functional connectivity density in hub regions**.

**Gray Matter Hub**	**White Matter Region**	**Correlation *r***	***P uncorrected***	***P FDR-corrected***
				
**YOUNGER**				
**Long-range Functional Connectivity Density**				
Inferior frontal R	Posterior thalamic radiation L	−0.29	0.001	
**Short-range Functional Connectivity Density**				
Angular R	Posterior corona radiata R	0.29	0.001	
**OLDER**				
**Long-range Functional Connectivity Density**				
Precuneus L	Splenium of corpus callosum	−0.35	< 0.001	
Precuneus L	Cerebral peduncle R	−0.34	< 0.001	
Precuneus L	Cerebral peduncle L	−0.35	< 0.001	
Precuneus L	Anterior limb of internal capsule R	−0.32	< 0.001	
Precuneus L	Anterior limb of internal capsule L	−0.33	< 0.001	
Precuneus L	Posterior limb of internal capsule R	−0.37	< 0.001	
Precuneus L	Posterior limb of internal capsule L	−0.36	< 0.001	
Precuneus L	Superior corona radiata R	−0.38	< 0.001	< 0.05
Precuneus L	Posterior corona radiata R	−0.40	< 0.001	< 0.05
Precuneus L	Cingulum cingulate gyrus R	−0.33	< 0.001	
Precuneus L	Superior longitudinal fasciculus R	−0.38	< 0.001	< 0.05
**Short-range Functional Connectivity Density**				
Precuneus L	Posterior corona radiata R	−0.32	< 0.001	

L, left; R, right. Correlation was controlled for age and sex. All results had uncorrected P < 0.001 and were further evaluated with False Discovery Rate (FDR) for corrections for multiple comparisons.

**Figure 4:**
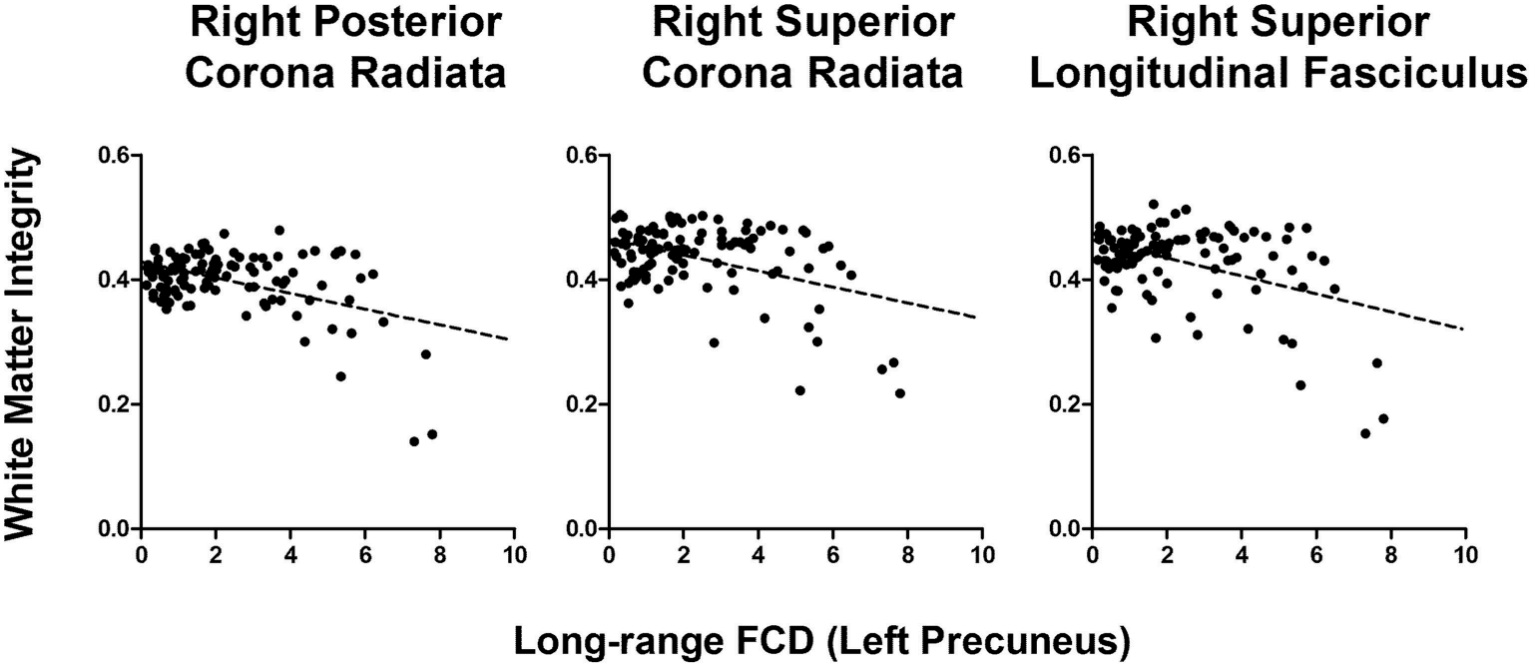
**Correlation graph between white matter integrity and long-range FCD in left precuneus in older adults**.

In older adults, long−range FCD in the left precuneus was correlated negatively to wide ranges of white matter tracts, including the splenium of the corpus callosum (*r* = −0.35), right and left cerebral peduncle (*r* = −0.34 and −0.35, respectively), right and left anterior limb of the internal capsule (*r* = −0.32 and −0.33, respectively), right and left posterior limb of the internal capsule (*r* = −0.37 and −0.36, respectively), superior and posterior corona radiata (*r* = −0.38 and −0.40, respectively), right cingulate gyrus (*r* = −0.33), and right superior longitudinal fasciculus (*r* = −0.38), whereas short−range FCD in the left precuneus was correlated positively to the right posterior coronal radiata (*r* = −0.32, all uncorrected *p* < 0.001). After FDR correction, long−range FCD in left precuneus was still significantly correlated with FA values in right posterior corona radiata, right superior corona radiata, and right superior longitudinal fasciculus (Figure 4).

### Relationship between cognitive function and neuroimaging measures

Cognitive measures (MMSE, digit forward and backward) were not significantly correlated to FCD measures in the younger group. For older adults, the digit span forward test was significantly positively correlated to long-range FCD in the left precuneus (*r* = 0.33) and short-range FCD in the left hippocampus (*r* = 0.36). MMSE was positively correlated to long-range FCD in the right angular gyrus (*r* = 0.30), and negatively correlated to the short-range FCD in the right postcentral gyrus (*r* = −0.33, all *p* < 0.001). No significant correlation was found between cognitive measures and white matter integrity in both groups.

## Discussion

Using a combined DTI and resting-state fMRI modalities, we investigated the association of aging with white matter integrity and resting-state functional connectivity, and their relationship in functional connectivity hub regions. Compared with the younger group, older adults showed more widespread reductions in white matter integrity, with selective preservation in brain stem tracts and the cingulum connected to the hippocampus and cingulate cortex, whereas short- and long-range FCD exhibited reduced FCD in the occipital, postcentral, and precentral cortex and increased FCD in DMN regions. In functional hub regions, older adults exhibited significantly increased FCD in the precuneus, posterior and middle cingulate, thalamus, hippocampus, fusiform gyrus, and inferior temporal cortex. Furthermore, the associations between white matter integrity and FCD in the functional hubs were identified primarily in older adults where short- and long-range FCD in the left precuneus was negatively correlated to the structural integrity of adjacent and nonadjacent white matter tracts, respectively. We also found that long-range FCD in the left precuneus was positively correlated to cognitive function. These findings suggest that the precuneus plays a crucial role in normal aging in the DTI-fMRI relationship and support the compensatory hypothesis of neurocognitive aging theory.

### Association of age with white matter integrity

Our findings of widespread reduced white matter integrity in older adults compared with younger adults are consistent with prior DTI reports (Pfefferbaum et al., 2000; Salat et al., 2005; Pagani et al., 2008; Heise et al., 2011). In addition, our observations of uneven age-associated decline in white matter integrity are consistent with prior reports (Pagani et al., 2008; Heise et al., 2011) that older adults had significant reductions in white matter integrity in the fornix, corpus callosum, and corona radiata. These affected white matter tracts involved projection, association, and commission fibers in the brain. Furthermore, we found that white matter integrity in the brain stem, cerebellum, and cingulum connected to the hippocampus and cingulate gyrus was relatively preserved in older adults. These preserved white matter regions were brainstem tracts and parts of association fibers (cingulum).

Our results suggest that normal aging is less associated with the integrity of white matter tracts that are related to vital body function, and intriguingly, the selective preservation of the cingulum in our healthy cohort may elucidate the differential effects of normal aging and neurodegenerative disease, such as Alzheimer's disease. The cingulum has been invariantly implicated in neurodegenerative disease (Rose et al., 2000; Zhang et al., 2007). The preservation of cingulum integrity in older adults and the absence of correlation between cognitive function and white matter integrity found in this study suggest that cognitive decline in normal aging is not driven by structural changes.

### Association of age with resting-state functional connectivity

Contrary to prior reports based on a public resting-state fMRI database (Tomasi and Volkow, 2012a), we found a diverse pattern of short- and long-range FCD in younger and older adults. Tomasi et al. reported that long-range FCD was negatively correlated to age in the posterior cingulate and ventral precuneus region, the core of DMN, and both short- and long-range FCD was positively correlated to age in the postcentral gyrus. By contrast, using the same FCD method, we found both increased short- and long-range FCD in DMN, and reduced FCD in the visual, somatosensory, and motor cortex.

The discrepancy in the results may be due to the predominance of younger subjects and the lack of older subjects (aged > 60) in the public database (1000 Functional Connectomes Project), which leads to statistical bias when applying Pearson's correlation to the relationship between age and FCD measures. However, a recent seed-based study revealed that resting-state connectivity in the posterior cingulate was negatively correlated with age in a continuous aging cohort (Mevel et al., 2013). Whereas, the reduced resting-state functional connectivity in DMN was the most common pattern reported in the elderly population (see Ferreira and Busatto, 2013 for reviews), numerous studies have revealed that age is positively correlated to functional connectivity in DMN areas (Meunier et al., 2009; Biswal et al., 2010; Toussaint et al., 2011). Furthermore, although the FCD method is capable of detecting DMN hubs and other functional networks, we found that our results of reduced FCD in visual, somatosenroy, and motor networks (except the cerebellum) in older adults differed from those reported by Tomasi and Volkow (2012a). Similarly, the inconsistency of the aging effect on these networks has also been reported previously (Wu et al., 2007; Allen et al., 2011).

Another reason for discrepancy of FCD results might be that our resting-state fMRI data were not pre-processed in the same way with Tomasi and Volkow (2012a). Tomasi and Volkow, for instance, regressed the global signal, which was deliberately not done in the present study to avoid introducing distortions in the time series data (Murphy et al., 2009; Anderson et al., 2011). Moreover, contrast to our single site neuroimaging data, the site effects (i.e., fMRI data collected from different MRI protocols and machines) in 1000 Functional Connectomes Project might also contribute the difference in FCD results.

### Association of cognitive function with neuroimaging measures

Decline in cognitive performance in old age is linked to both suboptimal neural processing in gray matter (Rajah and D'esposito, 2005) and reduced integrity of white matter (Madden et al., 2012). There have been a number of studies showing altered functional connectivity related to healthy aging and/or dementia, such as Alzheimer's disease, in which the later has been characterized as a connectivity disease (Van Den Heuvel and Hulshoff Pol, 2010; Hafkemeijer et al., 2012; Salami et al., 2014). For example, age-related disruption in cortico-hippocampal functional connectivity could lead to a more functionally isolated hippocampus at rest, suggesting aberrant hippocampal decoupling and deficits during mnemonic processing (Salami et al., 2014).

We did not find the correlation between cognitive function and the integrity of structural connectivity as measured by FA values. However, we did find positive correlations between FCD and digit span tasks/MMSE in precuneus, hippocampus, or angular gyrus in older group, suggesting increased resting-state connectivity in major functional hub is associated with preserved cognitive functions. Conversely, we found a negative correlation between MMSE and FCD in post-central gyrus, a region typically being activated during cognitive tasks and may reflect and anti-correlation between resting-state and salience network.

### Implication of the DTI-fMRI relationship in normal aging

Previous reports (Persson et al., 2006; Madden et al., 2007) have primarily reported DTI-fMRI relationships in older adults, showing negative correlations between FCD and white matter integrity. A recent study shows that increased connectivity in the compensatory network correlates positively with preserved white-matter integrity in bilateral fronto-parietal tracks (Burianova et al., 2015). Another study shows that greater standard deviation (SD) of BOLD signal was associated with better fluid abilities and memory and older adults with greater white matter integrity in all major white matter tracts had also greater SD of BOLD signal and better performance on tests of memory and fluid abilities (Burzynska et al., 2015). Marstaller et al. shows that age-related decline in white matter integrity and gray matter volume is associated with activity in prefrontal nodes of the salience and fronto-parietal network, possibly reflecting compensatory mechanisms (Marstaller et al., 2015). Collectively, these findings suggest the importance of structural integrity and functional connectivity in working memory performance associated with healthy aging.

Several neurocognitive aging theories have been proposed in relation to the DTI-fMRI relationship, including compensation, neural efficiency, nonselective overrecruitment, or underrecruitment (Bennett and Rypma, 2013). Our results supported compensation theory, that while normal aging was strongly associated with reduced structural integrity, the preservation of white matter tracts in the cingulum and increased FCD in DMN areas may help to compensate for the aging-related structural impairment and maintain healthy congitive function.

Compensatory increases in functional connectivity may be triggered to recruit additional neuronal resources when available structural networks, such as white matter integrity, are diminished (Reuter-Lorenz and Cappell, 2008; Park and Reuter-Lorenz, 2009; Cabeza and Dennis, 2012). This notion can be further supported by our results that older adults showed a positive correlation between cognitive assessments and FCD in DMN regions, and the predominantly negative correlations between FCD in the left precuneus and white matter intergrity. We did not find a significant DTI-cognitive or fMRI-cognitive correlation in younger people, and identified only two DTI-fMRI associations (Table 4). Thus our results support neither compensatory nor other neurocognitive mechanisms in younger people.

The structural integrity is expected to influence neural activity in the connected gray matter areas (i.e., at the terminals of the white matter tracts) compared to other gray matter regions to which they are not connected or only indirectly connected (Logothetis et al., 2001; Bennett and Rypma, 2013). Prior studies show that younger adult has positive correlation between white matter integrity and fMRI activation in adjacent gray matter regions (Toosy et al., 2004; Forstmann et al., 2008; Kim and Whalen, 2009; Van Eimeren et al., 2010) or partially adjacent gray matter region (Koch et al., 2010). In contrast, DTI–fMRI correlations were mostly negative for studies examining white matter integrity and fMRI activation in non-adjacent brain regions (Baird et al., 2005; Persson et al., 2006; Madden et al., 2007; Putnam et al., 2008; De Chastelaine et al., 2011).

Structural connectivity can influence the strength of functional connectivity (Damoiseaux and Greicius, 2009; Honey et al., 2010). Therefore, short- or long-range FCD is likely to be influenced by the integrity of white matter tracts. Indeed, we found that white matter integrity was correlated negatively with long-range FCD mostly in older people, and the DTI-fMRI relationship was associated with spatial proximity between the functional hubs and white matter tracts (Figure 3). When considering the negative correlation of DTI-fMRI in older adults, the association between the range of FCD and spatial proximity of white matter tracts further strengthens the compensatory hypothesis and helps to clarify the inconsistent findings of previous studies.

## Limitations

This study was subject to certain limitations. First, we examined neither the effect of sex, nor the interaction effect of sex and age on the between-group difference of neuroimaging measures, because the sex effect was not the primary focus of this study. However, we did control the effects of age and sex on FCD-FA correlations. Prior studies show that female participants exhibit greater overall cortical connectivity and more efficient local and global cortical networks than male subjects (Gong et al., 2009, 2011; Tomasi and Volkow, 2012b). The association of gender with functional connectivity and structural integrity is warranted to be investigated in the future based on a larger aging sample. Second, the FCD method quantified only the density of functional connections, but discarded the spatial connectivity patterns. However, when studying the DTI-fMRI relationship, this approach can greatly simplify the dimension of complex brain networks and provide a broader view of DTI-fMRI relationship. Third, the ROI approach may lower the strength of the DTI-fMRI correlation (Table 4; *r*-square 10–16%). Nevertheless, our results provide a starting point for investigating the detailed DTI-fMRI relationship using specific seed-based analyses. We note that the DTI-fMRI relationship may be nonlinear (Figure 4), which warrants additional study to model the DTI-fMRI relationship using nonlinear regression. Finally, the BOLD signal is an indirect measure of neural activity and is heavily influenced by the efficiency of neurovascular coupling. Aging may be associated with changes in cerebrovascular dynamics, which confounds the FCD analyses (Ferreira and Busatto, 2013). Furthermore, recent evidence has suggested that increased neural activity is not always associated with changes in cerebral blood flows (Huo et al., 2014); thus, our findings of DTI-fMRI association may overlook certain brain regions, such as the frontal cortex.

## Conclusions

This study comprehensively investigated the association of aging with the DTI-fMRI relationship by using a model-free analytic approach (i.e., FCD mapping and whole-brain white matter segmentation). Although normal aging was associated with widespread decline in white matter integrity and reduced visual, somatosensory, and motor functional connectivity, increased FCD in the DMN hub of the precuneus was associated with reduced white matter integrity and may be linked to the compensation of cognitive capacity in healthy elderly adults against the aging process.

## Author contributions

AY designed the study, analyzed image data, performed statistical results, and drafted the manuscript. ML and CH performed the experiment and collected the data. ST and CL designed the study and gave critical comments on the manuscript.

### Conflict of interest statement

The authors declare that the research was conducted in the absence of any commercial or financial relationships that could be construed as a potential conflict of interest.
